# Effects of dose rates on radiation-induced replenishment of intestinal stem cells determined by Lgr5 lineage tracing

**DOI:** 10.1093/jrr/rrv012

**Published:** 2015-04-01

**Authors:** Kensuke Otsuka, Toshiyasu Iwasaki

**Affiliations:** Radiation Safety Research Center, Nuclear Technology Research Laboratory, Central Research Institute of Electric Power Industry (CRIEPI), 2-11-1 Iwado-kita, Komae, Tokyo 201-8511, Japan

**Keywords:** dose-rate effects, tissue stem cell, Lgr5, low-dose rate

## Abstract

An understanding of the dynamics of intestinal Lgr5^+^ stem cells is important for elucidating the mechanism of colonic cancer development. We previously established a method for evaluating Lgr5^+^ stem cells by tamoxifen-dependent Lgr5-lineage tracing and showed that high-dose-rate radiation stimulated replenishment of colonic stem cells. In this study, we evaluated the effects of low-dose-rate radiation on stem cell maintenance. Tamoxifen (4OHT)-injected *Lgr5-EGFP-IRES-Cre^ERT2^ × ROSA-LSL-LacZ* mice were used, LacZ-labeled colonic crypts were enumerated, and the loss of LacZ^+^ crypts under low-dose-rate radiation was estimated. After 4OHT treatment, the number of LacZ-labeled Lgr5^+^ stem cells was higher in the colon of infant mice than in adult mice. The percentage of LacZ-labeled crypts in infant mice rapidly decreased after 4OHT treatment. However, the percentage of labeled crypts plateaued at ∼2% at 4 weeks post-treatment and remained unchanged for up to 7 months. Thus, it will be advantageous to evaluate the long-term effects of low-dose-rate radiation. Next, we determined the percentages of LacZ-labeled crypts irradiated with 1 Gy administered at different dose rates. As reported in our previous study, mice exposed to high-dose-rate radiation (30 Gy/h) showed a marked replenishment (*P* = 0.04). However, mice exposed to low-dose-rate radiation (0.003 Gy/h) did not exhibit accelerated stem-cell replenishment (*P* = 0.47). These findings suggest the percentage of labeled crypts can serve as a useful indicator of the effects of dose rate on the stem cell pool.

## INTRODUCTION

According to the ‘cell-of-origin in cancer’ model, many solid tumors originate from target cells that undergo an oncogenic event (i.e. genetic mutation) in the genome [1]. Ionizing radiation is a physical mutagen, and the mutation frequency increases in a dose-dependent manner. Epidemiological studies have shown that the incidence and mortality of solid cancers in Atomic Bomb survivors increased with the radiation dose, exhibiting a linear dose–response relationship [2, 3]. Thus, the incidence of radiation-induced cancer is likely associated with the total dose of radiation. However, an epidemiological study reported that the cancer mortality in individuals living in high background-radiation areas (HBRAs), where they receive extremely low-dose-rate radiation throughout their life, did not increase with the cumulative dose of radiation [4]. The discrepancies between these studies may therefore be explained by the dose rate rather than the overall dose.

‘Dose-rate effects’ are well-known responses in which the biological influence of low-dose-rate radiation is lower than that of the same dose of high-dose-rate radiation. Although evidence supporting the effects of dose rate have been reported elsewhere *in vitro* [5–18] and *in vivo* [19–21], most studies have focused on survival rates and cell death mechanisms, such as apoptosis. Several reports have shown that germline and somatic mutations arise in a dose-rate-dependent manner, with apparent dose-rate effects. In fish, germline mutations exhibit dose-rate effects between 0.0003 and 0.95 Gy/min [22]. Russell and Kelly demonstrated that mutations in the spermatogonia of mice exhibit dose-rate effects; however, the dose–response relationship exhibited the same linear slope from 0.0007 to 0.8 R/min [23]. This evidence suggests there is no dose-rate effect in this range. In somatic cells, which are associated with cancer incidence, mutations are induced in a dose-rate-dependent manner between 0.01 and 1.8 Gy/min [24]. Dose-rate effects have been observed in chromosomal aberrations between 1 and 20 mGy/day [25], a lower range than described previously [23]. These findings suggest the importance of considering the loss of cells carrying unstable chromosome aberrations during very long-term, low-dose-rate radiation exposure [26]. Tissue-level elimination of the abnormal cell-of-origin in cancer could play an important role in reducing the linear slope of the dose–response relationship [27]. Therefore, keeping the cell-of-origin intact is a possible mechanism of the dose-rate effects under extremely low-dose-rate radiation condition, such as in a HBRA.

The intestine is a major target organ in radiation-induced cancer [28]. The functional structure of the intestine consists of a monolayer of epithelial cells, all of which are produced by intestinal stem cells within the intestinal crypts. Intestinal stem cells expressing leucine-rich repeat-containing G-protein coupled receptor 5 (Lgr5) are cycling stem cells required for maintaining tissues in steady state, and they act as cells-of-origin in intestinal cancer [29]. However, the dose-rate effects in these stem cells have not been determined.

High-dose-rate whole-body radiation reduces the number of tissue stem cells by inducing cell death [30–32]. Even if the cells do not undergo cell death at the time of radiation, all of the tissue stem cells are damaged after high-dose-rate radiation. DNA damage can lead to aging, and exhaustion of the stem/progenitor cells in tissue stem cells [33, 34]. In addition to the cycling stem cells necessary to maintain steady state, tissues retain radioresistant, quiescent (or slow-cycling) stem cells for rescuing tissue reconstruction after drastic loss of cycling stem cells [35]. In fact, proliferation of slow-cycling stem cells is induced by high-dose irradiation [36]. Thus, replenishment of tissue stem cells by an undamaged stem-cell pool without stimulating proliferation of slow-cycling stem cells is important for tissue maintenance. There is no evidence that low-dose-rate whole-body radiation stimulates replenishment of tissue stem cells yet, although it is clear that low-dose-rate radiation does not significantly affect the proliferation rate of mammalian culture cells [37].

The conventional endpoints for cell death, such as apoptosis, only provide snapshots of biological phenomena and do not demonstrate the long-term, cumulative effects of radiation. As an alternative method, lineage-tracing systems have been used as tools for chasing target cells in tissues [38]. For the purposes of our research, we used *Lgr5-EGFP-IRES-Cre^ERT2^* × *ROSA26-LSL-LacZ* mice, which express Cre^ERT2^ in EGFP-expressing Lgr5^+^ stem cells [39]. In this model, administration of tamoxifen results in translocation of Cre^ERT2^-fused proteins into nuclei, and expression of the *LacZ* gene can be induced by Cre/loxP recombination. Once the *LacZ* gene is expressed, Lgr5^+^ stem cells and their daughter cells continuously express β-galactosidase, allowing visualization of Lgr5^+^ stem cells and their daughter cells in crypts. Loss of labeled crypts may indicate stimulation of stem-cell replacement. Using this technique, we showed that exposure to high-dose-rate radiation accelerated the replacement of murine colonic Lgr5^+^ stem cells [31]. We also found that the experimental design used in our previous work may not be applicable for the evaluation of low-dose-rate radiation because the number of labeled Lgr5^+^ stem cells decreases after tamoxifen injection alone, even in the absence of irradiation. Thus, proper experimental conditions are required for enabling retention of labeled crypts and evaluation of long-term cell retention.

In this study, we modified our experimental protocol to quantify stem-cell replenishment under low-dose-rate radiation and evaluated the effects of dose rates on Lgr5^+^ stem cell retention.

## MATERIALS AND METHODS

### Mice

*Lgr5-EGFP-IRES-Cre^ERT2^* knock-in mice (B6.129P2-*Lgr5^tm1(cre/ERT2)Cle^*/J; JAX mice #008875) and *ROSA26-LSL-LacZ* mice (B6.129S4-*Gt (ROSA)26Sor^tm1Sor^*/J; JAX mice #003474) were purchased from the Jackson Laboratory (ME, USA). The mice were bred in a conventional clean-room facility at the Central Research Institute of Electric Power Industry (CRIEPI) under conditions of controlled temperature (24 ± 2°C) and humidity (45% ± 5%), with a 12-h light–dark cycle and *ad libitum* access to γ-sterilized food (CLEA Japan, Tokyo, Japan) and filter-sterilized deionized water. All animal experiments were approved by the Animal Research and Ethics Committee at the CRIEPI and were performed in accordance with the guidelines for animal care in Japan.

### Irradiation

To irradiate mice with high-dose-rate (30 Gy/h) X-rays, an MBR-320R generator (Hitachi Ltd, Tokyo, Japan) installed in a conventional clean-room facility at the CRIEPI was operated at 260 kV, with a 4.5-mA tube current and a 0.5-mm Al + 0.3-mm Cu filter. ^137^Cs γ-rays (0.003 Gy/h) were used for long-term low-dose-rate irradiation at the low-dose-rate irradiation facility at the CRIEPI. The dose of irradiation was determined using a photoluminescence glass dosimeter GD-351 (Asahi Techno Glass Corporation, Tokyo, Japan). Mice were subjected to low-dose-rate γ-rays for ∼2 weeks (the cumulative dose was 1 Gy). Control mice were sham-irradiated and handled in the same manner as the test animals.

### β-Galactosidase (LacZ) staining for lineage tracing

Detailed methods for the lineage tracing of Lgr5^+^ cells have been described previously [31]. In brief, in order to stain LacZ-labeled crypts, 4-hydroxytamoxifen (4OHT, Sigma, St Louis, MO, USA) at 10 mg/ml (3 mg/40 g body weight) was intraperitoneally injected into *Lgr5-EGFP-IRES-Cre^ERT2^* × *ROSA26-LSL-LacZ* mice. One month after 4OHT administration, the mice were irradiated with X-rays or γ-rays. The mice were sacrificed 2 weeks after irradiation, and the intestinal LacZ^+^ crypts were counted as described [31]. In brief, the isolated distal colon was washed with phosphate-buffered saline (PBS), opened longitudinally, and placed on filter paper. The tissue samples were then fixed and stained to assess β-galactosidase activity [39]. The samples were then embedded in paraffin, sectioned horizontally, and counterstained with eosin (Sakura Finetek, Tokyo, Japan). Fully LacZ-labeled crypts were counted as LacZ^+^ crypts, and the percentage of LacZ^+^ crypts (*%LacZ crypts*) was calculated as follows:

*%LacZ^+^ crypts* = (number of fully LacZ-labeled crypts within the obtained images) × 100/(number of all crypts contained in the obtained images).

We counted at least 500 crypts in each mouse.

### Image analysis

We used ImageJ version 1.46r (National Institutes of Health) to determine the LacZ^+^ area in each crypt. We first split the images of the crypt sections stained with X-gal (blue) and eosin (pink) into three channels (RGB). We used the red and blue channels to determine the areas of the LacZ^+^ cells and whole crypts. The area was measured after determining the shapes of crypts or LacZ^+^ cells.

### Real-time reverse transcription polymerase chain reaction

Tissue samples of the whole distal colon were harvested, cut into 5-mm pieces, and immediately frozen in liquid nitrogen. The frozen pieces were pulverized using a TK-AM5-S bullet mill (Tokken Inc., Chiba, Japan). Total RNA was extracted, purified using an Ambion RNAqueous-4PCR kit (Thermo Fisher Scientific Inc., MA, USA), and reverse-transcribed into cDNA using Invitrogen SuperScript II RNase-H RT enzyme (Thermo Fisher Scientific Inc., MA, USA). Primers and TaqMan probes for the *Lgr5* gene (Assay ID: Mm00438890_m1) were purchased from Life Technologies. Gene expression levels were determined using an Applied Biosystems PRISM 7900HT analyzer (Thermo Fisher Scientific Inc.) and normalized to glyceraldehyde-3-phosphate dehydrogenase (*Gapdh*) (Assay ID: Mm99999915_g1).

### Statistical analyses

Student's *t*-tests were used to compare the frequencies of LacZ^+^ crypts following 4OHT injection into mice of different ages (1 and 4 weeks old) and to evaluate the radiation-induced replenishment of colonic Lgr5^+^ stem cells. Tukey–Kramer multiple comparison tests were used to evaluate gene expression.

## RESULTS

### Optimization of experimental conditions for evaluating long-term Lgr5^+^ stem-cell retention

**Fig. 1.**
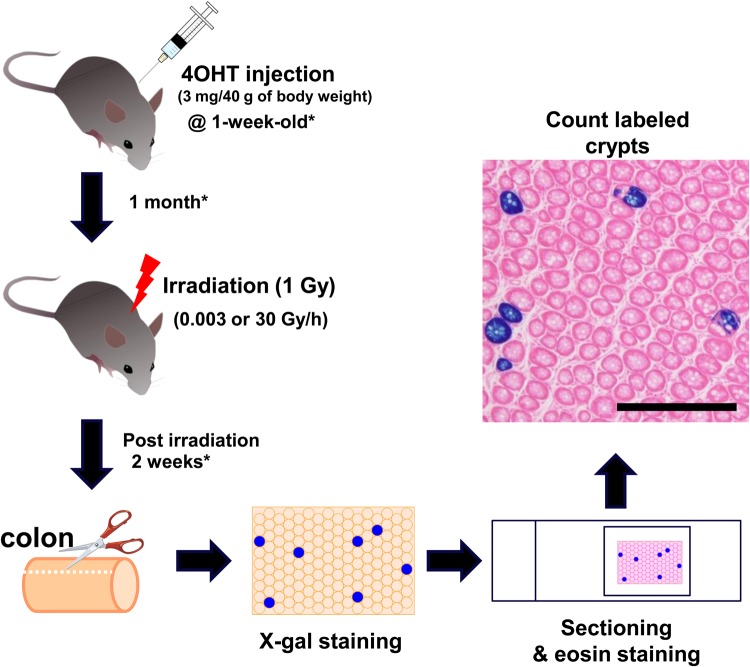
Diagram of stem cell replacement analysis. To trace labeled stem cells in colonic crypts, *Lgr5-EGFP-IRES-Cre^ERT2^* × *ROSA-LSL-LacZ* mice were injected with 4OHT (3 mg/40 g body weight). After 4OHT injection, mice were irradiated with 1 Gy at one of two dose rates (30 or 0.003 Gy/h). After irradiation, the colon was harvested and opened longitudinally, and the fragment of colonic tissue was stained in X-gal solution to visualize LacZ-labeled crypts. The fragment of the colon was then embedded in paraffin, sectioned and counterstained with eosin to obtain high-contrast samples. The number of LacZ-labeled crypts was counted and converted into a percentage. Scale bars: 500 μm. Asterisks denote the final experimental conditions determined by the results of Figs 2 and 3.

**Fig. 2.**
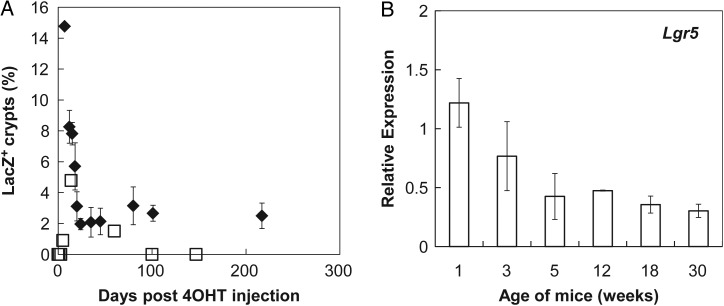
Effects of mouse age on LacZ-labeling efficiency. (**A**) The percentages of LacZ^+^ colonic crypts are shown. Data points were obtained from 7 to 217 days after 4OHT injection. The symbols denote the age of the mice at the time of 4OHT injection; 1-week-old mice (closed diamonds) and 4–7-week-old mice (open squares) are shown. (**B**) Expression of the *Lgr5* gene in the colon of mice at different ages was quantified by real-time reverse transcription polymerase chain reaction (RT-PCR). Gene expression was normalized to the expression level of the *Gapdh* gene (*n* = 2–5).

A schematic diagram of the experiment is shown in Fig. 1. We administered 4OHT to *Lgr5-EGFP-IRES-Cre^ERT2^* × *ROSA-LSL-LacZ* mice and then exposed the mice to radiation at different dose rates (0.003 or 30 Gy/h). After radiation exposure, staining and sectioning, LacZ-labeled crypts were counted. Note that not all colonic crypts of 4OHT-treated *Lgr5-EGFP-IRES-Cre^ERT2^* × *ROSA-LSL-LacZ* mice were labeled (Fig. 1) [31, 40]. Labeling efficiency in the crypts was evaluated by determining the decrease in the number of labeled Lgr5^+^ stem cells during cell replacement. The efficiency of LacZ-labeling upon 4OHT injection in mice of different ages has not been previously investigated. Therefore, we first determined the percentage of LacZ^+^ crypts in 4OHT-treated infant (1-week-old) and young adult (4-week-old) mice. Prior to 2 weeks after 4OHT injection, the percentage of LacZ-labeled colonic crypts reached a peak, and the labeling frequency was significantly higher in infant mice than in young adult mice (Fig. 2A). We also investigated the gene expression levels of *Lgr5* in the whole mouse colons at different ages and confirmed that *Lgr5* expression was significantly higher in 1-week-old mice than in mice of other ages (Fig. 2B).

At 2 weeks after 4OHT injection, the percentage of LacZ-labeled crypts gradually decreased. This may have been a result of spontaneous cell turnover in the tissue due to stem cell competition caused by a neutral drift between labeled and unlabeled stem cells [41, 42]; alternatively, this observation may be explained by age-dependent crypt fission [43]. Next, we administered 4OHT to infant (1-week-old) and adult (4–7-week-old) mice and chased the percentage of LacZ-labeled crypts to compare the time-course of LacZ^+^ crypt appearance. In the adult group, labeled colonic crypts were observable until 100 days after administration and became undetectable thereafter. In contrast, the infant group maintained a constant percentage of labeled crypts up to 7 months (Fig. 2A).

**Fig. 3.**
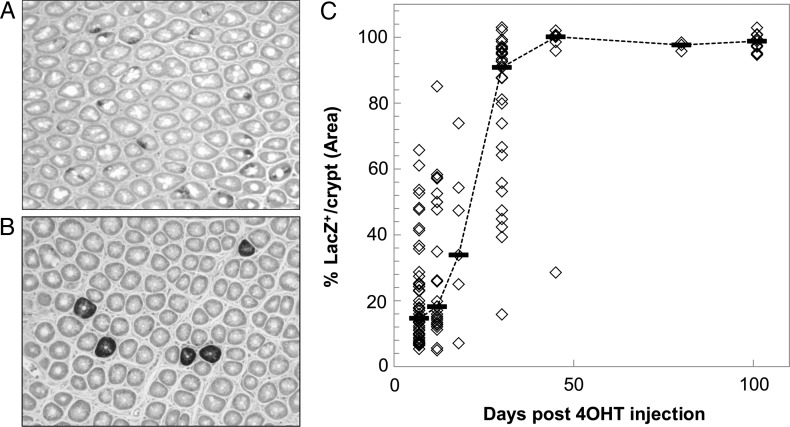
Expansion of LacZ-labeled cells in colonic crypts after 4OHT injection. One-week-old mice were injected with tamoxifen (4OHT) and then sacrificed 1 month after treatment. Sections of colonic crypts were examined 7 and 30 days after 4OHT injection, as shown in (**A**) and (**B**), respectively. (**C**) The LacZ^+^ area (in percentage) per crypt at 7, 12, 18, 30, 45, 80 and 101 days after 4OHT injection. Each diamond denotes one crypt. Dashed bars denote the medians. All data points were obtained using ImageJ software.

To elucidate the patterns of cellular competition among LacZ-labeled and unlabeled cells in each crypt, we quantified the LacZ^+^ area in each crypt by image analysis at several time-points after 4OHT injection. One week after 4OHT injection, numerous crypts contained LacZ^+^ cells in infant mice. However, the area of LacZ^+^ cells per crypt was small (Fig. 3A). Although the percentage of LacZ^+^ crypts subsequently decreased, almost all of the cells were LacZ^+^ in a labeled crypt at several months after 4OHT injection (Fig. 3B). The median LacZ-labeled areas per crypt were 14.7% at Day 7 and 90.8% at Day 30 after 4OHT injection (Fig. 3C). The area of the crypt was fully labeled with LacZ 45 days after 4OHT injection. The number of fully labeled crypts remained unchanged thereafter (Figs 2A and 3C).

Based on these observations, we established a new protocol for evaluating the long-term retention of colonic Lgr5^+^ stem cells, as follows: (i) administer 4OHT to infant mice; (ii) irradiate the mice 1 month after 4OHT administration, and (iii) evaluate LacZ^+^ crypts 2 weeks after the end of irradiation (Fig. 1).

### Effects of dose rates on the replenishment of colonic Lgr5^+^ stem cells

Next, we compared the replenishment rate of Lgr5^+^ stem cells induced by 1 Gy of either high-dose-rate X-rays (30 Gy/h) or low-dose-rate γ-rays (0.003 Gy/h). The results are shown in Table 1. In the high-dose-rate group, 1.40% ± 0.36% of the crypts were LacZ^+^ in irradiated mice (*n* = 7), whereas 1.98% ± 0.55% of the crypts were LacZ^+^ in non-irradiated control mice (*n* = 6). The percentage of LacZ^+^ crypts in mice treated with high-dose-rate irradiation was significantly lower than that in the non-irradiated control group (*P* = 0.04). These results were consistent with our previous results [31]. For mice treated with low-dose-rate radiation, 1.78% ± 0.73% of crypts were LacZ^+^ (*n* = 15), whereas 2.05% ± 0.79% of crypts in non-irradiated control mice were LacZ^+^ (*n* = 6), a non-significant difference (*P* = 0.47).

**Table 1.** Percentage of colonic LacZ-labeled crypts after 1 Gy of high-dose-rate (30 Gy/h) or low-dose-rate (0.003 Gy/h) irradiation

Dose-rate (Gy/h)	Dose (Gy)	Number of crypts counted	Number of LacZ^+^ crypts (fully labeled)	% LacZ^+^ crypts	% LacZ^+^ crypts (mean ± S.D.)	*P*-value (*t*-test)
30	sham	1591	21	1.32	1.98 ± 0.55	0.04
		1739	38	2.19		
		1353	37	2.73		
		4086	78	1.91		
		2950	68	2.31		
		2631	37	1.41		
	
	1	1948	22	1.13	1.40 ± 0.36	
		3443	48	1.39		
		3495	69	1.97		
		2511	30	1.19		
		2179	24	1.10		
		3961	47	1.19		
		1849	34	1.84		

0.003	sham	762	17	2.23	2.05 ± 0.79	0.47
		966	13	1.35		
		804	12	1.49		
		652	23	3.53		
		2100	38	1.81		
		1693	32	1.89		
	
	1	943	10	1.06	1.78 ± 0.73	
		1579	27	1.71		
		2534	28	1.10		
		1707	35	2.05		
		2359	31	1.31		
		3213	29	0.90		
		2829	59	2.09		
		2050	35	1.71		
		1866	56	3.00		
		1561	20	1.28		
		2078	33	1.59		
		1693	55	3.25		
		1349	38	2.82		
		3636	42	1.16		
		2614	44	1.68		

## DISCUSSION

In this study, we attempted to develop a method for detecting replenishment of the colonic Lgr5^+^ stem cell pool of animals exposed to radiation at different dose rates. To this end, we first determined the conditions under which LacZ-labeled stem cell numbers were maintained in the stem cell pool. We found that two distinct time-points were important: (i) the age of the mice at the time of 4OHT injection, and (ii) the age of the mice at the time of assay.

In 4OHT-injected infant mice, the population of LacZ-labeled colonic crypts remained stable from 1 to 7 months post-treatment. In contrast, in 4OHT-injected adult mice, the number of LacZ-labeled crypts gradually decreased post-treatment. The accuracy of these results depends on the efficiency of LacZ-labeling, and the labeling efficiency depends on the gene expression level of *Lgr5* in the intestinal cells. Dehmer *et al.* reported that the number of EGFP^+^ cells in *Lgr5-EGFP-cre^ERT2^* mice does not change with age, whereas the level of *Lgr5* expression in the intestines of 14-day-old mice is higher than that of 4-week-old adult mice [44]. Here, we confirmed that *Lgr5* expression was ∼3.5-times higher in 1-week-old mice than in older (>3-week-old) mice (Fig. 2B). Thus, the number of labeled crypts detected may be dependent on the number of Lgr5^+^ stem cells labeled in each crypt.

The age of the mice at the time of assay was also an important factor because the percentage of LacZ-labeled crypts after 4OHT injection is influenced by the number of labeled and unlabeled stem cells [41, 42] and by tissue dynamics, such as crypt fission [43]. Image analyses of samples obtained 7 days after 4OHT injection showed that more than half of the crypts had only 15% or less LacZ-labeled area per crypt. Thus, the LacZ-labeled cells in these crypts were the minority and may be stochastically eliminated from crypts. This rapid decrease in LacZ-labeled crypts could be caused by an age-dependent difference in the rate of crypt fission. Cheng and Bjerknes observed that the percentage of crypt fission in the colons of 1-week-old mice was ∼35%, whereas that in 4-week-old adult mice was 5–10% [43]. Our data indicated that the number of LacZ-labeled colonic crypts decreased rapidly in 4OHT-treated 1-week-old mice.

We were unable to distinguish crypt fission from other causative factors for stem cell pool reduction in infant mice irradiated immediately after 4OHT injection. To address this issue, experiments were conducted at several time-points to determine the time at which crypt fission primarily influenced the LacZ-labeled crypts. The data indicated that more than half of each crypt was occupied with LacZ-labeled cells (>90% of each crypt area) 30 days after 4OHT injection (Fig. 3C). These data showed that crypt fission may no longer be the primary influential factor for the emergence of LacZ-negative crypts. The percentage of LacZ-labeled crypts was constant from 30 days to over 200 days after 4OHT injection (Fig. 2A). Therefore, the mice were irradiated 30 days after 4OHT injection to quantify the loss of Lgr5^+^ stem cells in the colon. We confirmed that radiation accelerated the loss of LacZ-positive cells in the colonic crypts and finally established a method for evaluating the robustness of the Lgr5^+^ stem cell pool by enumerating fully labeled crypts that persisted after radiation exposure.

Biological endpoints such as apoptosis rates and DNA repair kinetics can be used for evaluating radiation sensitivity and for characterizing the mechanisms underlying cellular replacement. However, it is difficult to compare those endpoints under several dose-rate conditions because of the variation in the period of exposure. The percentage of LacZ-labeled crypts reflects a tissue-level endpoint, which includes cellular biological processes. We found that our endpoint can be a useful indicator for evaluating dose-rate effects on stem-cell replenishment. We previously showed that the size of the Lgr5^+^ stem cell pool was quickly reduced under high-dose (high-dose-rate) radiation exposure [31].

Lost Lgr5^+^ stem cells may be replaced with radio-resistant, quiescent, or slow-cycling stem cells, such as Bmi-1^+^ or mTert^+^ cells [35, 36], or by the dedifferentiation of transit amplifying (TA) cells, such as Dll^+^ cells [45]. In this study, we could not detect the dedifferentiation of TA cells into Lgr5^+^ cells because mice were exposed to radiation 1 month after 4OHT injection, at which point almost all Lgr5^+^ (EGFP^+^) stem cells and TA cells were already labeled with LacZ. Therefore, we considered that the lost LacZ-labeled Lgr5^+^ stem cells should have been replaced by slow-cycling stem cells in the same crypt to recover the colonic Lgr5^+^ stem cell pool. A recent study demonstrated that Lgr5^+^ stem cells are indispensable for the repopulation of the Lgr5^+^ stem cell pool after radiation exposure [46]. Presumably, this evidence does not refute the hypothesis that Lgr5^+^ stem cells are derived from slow-cycling stem cells because slow-cycling stem cells are weakly expressing the *Lgr5* gene, and those slow-cycling stem cells can be completely lost from crypts by Lgr5^+^ abrasion. Thus, these slow-cycling Lgr5^+^ stem cells may be able to repopulate the Lgr5^+^ stem cell pool if they can survive after high-dose radiation exposure.

Replenishment of a stem-cell pool by slow-cycling stem cells after high-dose radiation exposure could lead to initiation of carcinogenesis because it allows fixation and expansion of damaged stem cells in the tissues. In the case of low-dose-rate radiation, we could not detect replenishment by slow-cycling stem cells. These findings suggest that stem cells that have low levels of DNA damage result in improved cancer prevention because abnormal stem cell clones are removed and the reserve of undamaged stem cells is sufficient to compensate for a depleted stem-cell pool [47]. Under such low-dose-rate irradiation conditions, we should take into account cell competition in a stem-cell pool and whether mutated stem cells would be eliminated from tissues during extremely low-dose-rate radiation exposure in future studies.

## FUNDING

This work was supported by a Grant-in-Aid for Scientific Research (No. 23651051) from the Japan Society for the Promotion of Science (JSPS) and by a grant from the Study (Group) of the Health Effects of Radiation Organized by the Ministry of the Environment, Japan. Funding to pay the Open Access publication charges for this article was provided by CRIEPI.
